# Associations between dynapenia, cardiovascular hospitalizations, and all‐cause mortality among patients on haemodialysis

**DOI:** 10.1002/jcsm.13039

**Published:** 2022-08-02

**Authors:** Shun Yoshikoshi, Shohei Yamamoto, Yuta Suzuki, Keigo Imamura, Manae Harada, Shiwori Osada, Kentaro Kamiya, Atsuhiko Matsunaga

**Affiliations:** ^1^ Department of Rehabilitation Sciences Kitasato University Graduate School of Medical Sciences Kanagawa Japan; ^2^ Department of Epidemiology and Prevention Center for Clinical Sciences, National Center for Global Health and Medicine Tokyo Japan; ^3^ Department of Rehabilitation Sagami Circulatory Organ Clinic Kanagawa Japan; ^4^ Department of Nephrology Tokyo Ayase Kidney Center Tokyo Japan

**Keywords:** Dynapenia, Handgrip strength, Haemodialysis, Mortality, Quadriceps isometric strength

## Abstract

**Background:**

Low muscle strength is associated with adverse clinical outcomes in patients undergoing haemodialysis (HD). No studies have reported the association between dynapenia, defined by both low handgrip strength (HGS) and quadriceps isometric strength (QIS), and long‐term clinical outcomes in patients on HD. We examined the associations between dynapenia, cardiovascular (CV) hospitalizations, and all‐cause mortality in the HD population.

**Methods:**

This retrospective study used data from outpatients undergoing HD at two dialysis facilities between October 2002 and March 2020. We defined low muscle strength as an HGS of <28 kg for men and <18 kg for women and a QIS of <40% dry weight. Furthermore, we categorized dynapenia into three groups: robust (‘high HGS and high QIS’), either low HGS or low QIS (‘low HGS only’ or ‘low QIS only’), and dynapenia (‘low HGS and low QIS’). The outcomes were all‐cause mortality and a composite of CV hospitalizations and mortality. Cox proportional hazards and negative binomial models were used to examine these associations.

**Results:**

A total of 616 patients (mean age, 65.4 ± 12.2 years; men, 61%) were included in the analyses. During the follow‐up (median, 3.0 years), a total of 163 deaths and 288 CV hospitalizations occurred. Patients with the either low HGS or low QIS [hazard ratio (HR), 1.75; 95% confidence intervals (CIs), 1.46–2.10] and dynapenia (HR, 2.80; 95% CIs, 2.49–3.14) had a higher risk of mortality than those in the robust group. When compared with the robust group, the either low HGS or low QIS [incidence rate ratio (IRR): 1.41, 95% CI: 1.00–1.99] and dynapenia (IRR: 2.04, 95% CI: 1.44–2.89) groups were associated with a significantly higher incident risk of CV hospitalizations.

**Conclusions:**

Dynapenia (muscle weakness in both upper and lower extremities) was associated with increased risks of all‐cause mortality and CV hospitalizations among patients on HD. Screening for dynapenia using both HGS and QIS may be useful for prognostic stratification in the HD population.

## Introduction

Dynapenia is a physical condition defined as an age‐associated loss of muscle strength^1^, ^2^; it is considered to be different from sarcopenia, which is defined as the loss of both muscle strength and muscle mass.^2^ Growing evidence has shown that low muscle strength is associated with a higher risk of mortality, whereas low muscle mass is not consistently associated with mortality in the older population.^3^ These results are also observed in population with chronic disease,^4^, ^5^ particularly end‐stage renal disease (ESRD).^6^, ^7^ These results suggest the importance of in‐depth assessment of muscle strength in these populations.

A growing body of literature on patients undergoing haemodialysis (HD) suggests that low muscle strength, such as handgrip strength (HGS) or quadriceps isometric strength (QIS), is associated with a higher risk of all‐cause mortality.^8^, ^9^ For example, a meta‐analysis of patients undergoing dialysis demonstrated that a 1‐kg unit decrease in HGS was associated with a higher risk of mortality.^8^ Moreover, a prospective cohort study showed that patients with low QIS had higher mortality rates than those with high QIS.^9^

Some issues regarding the implications of dynapenia among patients on dialysis remain. First, no studies have defined dynapenia in both low HGS and low QIS. Some studies have indicated that the correlation between HGS and QIS is low.^10^, ^11^ Although HGS is a clinically useful tool to measure muscle strength, physical performance, such as walking ability and balance function, is mainly dependent on lower extremity muscle strength. Therefore, to assess muscle strength in more detail, assessing strength in both the upper and lower limbs using HGS and QIS is recommended.^1^ Dynapenia, which is defined as both HGS and QIS, has been described as a prognostic factor for all‐cause mortality among patients with cardiovascular (CV) disease.^12^ Second, few studies have reported an association between muscle strength and multiple CV events (i.e. the number of first and recurrent incident events).^13^ Patients on HD are more likely to undergo multiple CV hospitalizations,^14^ and these recurrent hospitalizations are associated with a higher mortality risk.^15^ Therefore, it is necessary to consider CV hospitalizations as multiple events in this population.

To address the aforementioned issues, this study aimed to investigate whether dynapenia, defined as both low HGS and QIS, was associated with higher risks of all‐cause mortality and CV hospitalizations among patients undergoing HD.

## Methods

### Study setting

The study was carried out in two HD clinics in Kanagawa and Tokyo, Japan. These clinics annually evaluate patients' physical performance, as a part of their disease management programme.^16^ These data are stored in the medical records of each clinic. All participants provided written informed consent to use these existing data for research when the patient first started dialysis treatment at each facility. This study was approved by the Institutional Review Board/Ethics Committee of Kitasato University of Allied Health Sciences (approval number: 2017‐026B‐2) and conducted in accordance with the principles of the Declaration of Helsinki.

### Analytic cohort

In this study, we included outpatients who received maintenance HD treatment (at least 3 times per week for at least 3 months) and had results of dynapenia assessments between October 2002 and March 2020. Patients who provided written consent for the use of existing data but did not undergo dynapenia assessment (e.g. due to scheduling conflicts or refusal to participate in the assessment) were excluded from the analysis. Although these centres attempt to assess dynapenia in all maintenance HD outpatients, evaluation could not be conducted for some patients due to two main reasons: (1) patients with unstable medical conditions (e.g. recent myocardial infarction or angina pectoris, uncontrolled cardiac arrhythmias, haemodynamic instabilities, uncontrolled hypertension, renal osteodystrophy with severe arthralgia, or severe dementia). These patients might not be able to tolerate the cardiac load during the maximal muscle strength measurement and could be at risk for worsening of their condition. (2) Those who had been hospitalized within 3 months before the assessment, because they were more likely to experience a functional decline due to worsening medical conditions, which could have worsened their physical function.^17^ Patients admitted to the hospital within 3 months before the evaluation were evaluated and included in this study if their condition was stable after at least 3 months.

### Dynapenia assessment

**Figure 1:**
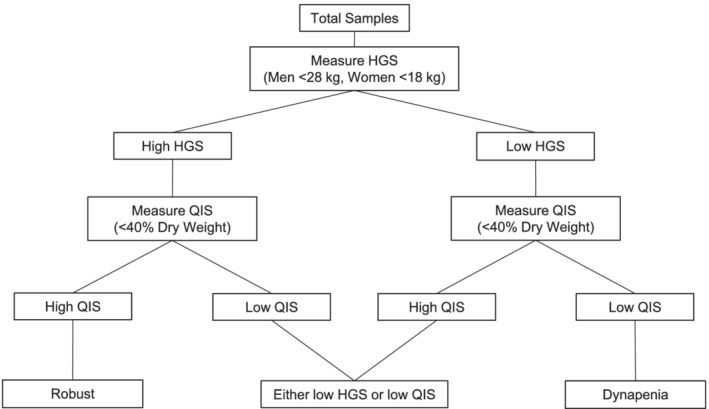
Flow diagram of patients divided into three groups. HGS, handgrip strength; QIS, quadriceps isometric strength.

Muscle strength was assessed using HGS and QIS. The HGS and QIS of most patients were measured prior to dialysis. However, those who could only be assessed on post‐dialysis or non‐dialysis days were evaluated when they were in good physical condition. The HGS was measured using a digital dynamometer (TKK 5101 Grip‐D; Takei, Tokyo, Japan). Maximal isometric grip force was collected for both hands, for 3 s each, with the patient in the sitting position and with the elbow joint angle fixed at 90° flexion. The HGS was measured twice per hand, and the average of the highest value from each hand expressed as the absolute value (kg) was used in the analyses. According to the Asian Working Group for Sarcopenia 2019 criteria, low HGS was defined as HGS < 28 kg for men and <18 kg for women.^18^ The QIS was evaluated using a handheld dynamometer (μtas F‐1; Anima, Tokyo, Japan). Patients were asked to sit on a bench with their hip and knee joint angles fixed at 90° flexion. Thereafter, the maximum voluntary isometric knee extensor strength was measured three times. Maximum QIS was expressed as a percentage of dry weight (DW), that is, the average of the right and left maximum isometric leg strength divided by DW (% DW). While there are no clear cut‐off values for QIS, we defined low QIS as <40% DW according to a previous study conducted among patients undergoing HD.^9^ We defined the baseline point as the first time the patient's muscle strength (HGS and QIS) was evaluated. As there are no gold standard algorithms for diagnosing dynapenia, we classified participants into three categories as follows: robust (‘high HGS and high QIS’), either low HGS or low QIS (‘low HGS and high QIS’ or ‘high HGS and low QIS’), and dynapenia (‘low HGS and low QIS’) (*Figure* 1).^1^

### Covariates

Demographic factors (age, sex, and HD vintage) and physical constitution [height, DW, body mass index (BMI), primary kidney disease (diabetes, glomerulonephritis or cystic kidney disease, hypertension, unknown or other), comorbidity index score, serum albumin, serum haemoglobin, serum creatinine, and C‐reactive protein (CRP)] were collected from the medical records at baseline. A comorbidity score, developed for patients on dialysis, which comprised any of the following was used to quantify comorbidity in this study: the primary causes of ESRD, atherosclerotic heart disease, congestive heart failure, cerebrovascular accident/transient ischaemic attack, peripheral vascular disease, dysrhythmia, other cardiac diseases, chronic obstructive pulmonary disease, gastrointestinal bleeding, liver diseases, cancer, and diabetes.^19^ As physical activity, the number of steps was measured using an accelerometer (Lifecorder; Suzuken Co., Ltd., Nagoya, Japan). Patients wore the accelerometer around their waist and were instructed to wear it continuously during their waking hours for 7 days. They were also asked to maintain their usual lifestyle routines. Measurement of the average number of steps on four non‐dialysis days was used in the analysis.^20^

### Outcomes

The primary outcome was all‐cause mortality and the secondary outcome was the composite of multiple CV hospitalizations and/or all‐cause mortality. Death was incorporated into the analyses by treating it as an additional event.^21^ Participants were followed up from baseline to the event occurrence date: death, transfer, change in renal replacement therapy modality, loss of follow‐up, or the end date of the study follow‐up (March 2020). All‐cause death and CV hospitalizations were collected from the medical records of the facilities. CV hospitalization was defined using hospitalization codes as follows: (1) hospitalization associated with a diagnosis coding for angina, acute myocardial infarction, cardiac arrest/sudden death, congestive heart failure, cardiomyopathy, valvular heart disease, atrial fibrillation, other arrhythmias, pericarditis and/or tamponade, and other cardiac or CV diseases; or (2) hospitalization associated with a procedure coding for coronary angioplasty, coronary artery bypass graft, valve repair or replacement, pacemaker placement, or pericardial procedure.

### Treatment of missing data

The number of missing data was as follows: serum albumin (*n* = 2), serum haemoglobin (*n* = 4), serum creatinine (*n* = 4), CRP (*n* = 34), primary causes of ESRD (*n* = 2), comorbidity index (*n* = 2), and physical activity (*n* = 165).

We performed multiple imputations using the chained equations method, assuming that the analysed data were missing at random.^22^ Results from 20 imputed datasets were combined for analysis using Rubin's formula. The imputation used all the variables involved in all the analytic models, including the facility variable and the outcome variables of time‐to‐event and event status. To fill in missing values, the predictive mean matching imputation method was used for the comorbidity index, serum albumin, serum haemoglobin, serum creatinine, CRP, and physical activity. Multinomial logistic regression imputation was used for the primary kidney disease of ESRD.

### Statistical analysis

We calculated person‐years of follow‐up for each participant from the baseline to the event occurrence date. To investigate whether HGS or QIS alone was associated with all‐cause mortality, we conducted Cox proportional hazards regression to calculate hazard ratios (HRs) and 95% confidence intervals (CIs). Negative binomial regression models were used to estimate the incidence rate ratio (IRR) and 95% CIs for CV hospitalizations. We analysed these associations by treating HGS and QIS as continuous or categorical exposure terms, respectively. In these analyses, continuous variables were standardized to compare the prognostic abilities of HGS and QIS. To visualize these associations, we used restricted cubic splines with 3 knots placed at the 5th, 50th, and 95th percentiles as recommended.^23^ *P* for linearity was tested by examining the association of HGS and QIS (as continuous terms) with all‐cause mortality and CV hospitalizations in the adjusted Cox and binomial regression models.

We used the Kaplan–Meier method to visualize the cumulative survival curve for all‐cause mortality between the robust, either low HGS or low QIS, and dynapenia groups. Differences in survival between the groups were evaluated using the log‐rank test. We used Cox proportional hazards regression for the association between dynapenia and all‐cause mortality. To estimate the associations with the multiple CV hospitalizations, we used negative binomial regression models. In both Cox proportional hazard regression and negative binomial regression models, we adjusted for age, sex, height, DW, HD vintage, comorbidity index, serum albumin, serum haemoglobin, serum creatinine, and CRP levels. All covariates were known to be factors of adverse outcomes among HD patients.^24^, ^25^ Cluster effects at the facility level were accounted for using robust variance estimates. To investigate the associations of dynapenia with the outcomes, we used trend tests in the Cox proportional hazard regression analyses and negative binomial regression analyses by treating categorical variables (robust, low HGS or QIS, and dynapenia) as continuous variables.

Furthermore, two sensitivity analyses were conducted. First, assuming that physical activity is a mediator of low muscle strength,^26^ we additionally adjusted physical activity to investigate the association between dynapenia and outcomes. Second, because the range of HGS and QIS distributions differed between men and women (Supporting Information, *Figure* *S1*), we investigated the associations of HGS, QIS, and dynapenia with all‐cause mortality and CV hospitalizations stratified according to sex.

The proportional hazards assumption was assessed on the basis of the analysis of Schoenfeld residuals, and no violations of proportionality were observed.

Statistical significance was defined as a two‐tailed *P*‐value < 0.05. Statistical analyses were performed using Stata, Version 16.0 (StataCorp LLC, College Station, TX, USA).

## Results

### Patients' characteristics

A total of 828 outpatients were receiving maintenance HD treatment from October 2002 to March 2020. Of these, 171 patients who could not participate in the assessment programme of physical function due to the following reasons were excluded: 21 patients hospitalized within 3 months before the evaluation, 72 were clinically unstable, 72 refused to participate in this study, and 6 had other reasons. Finally, 657 patients participated in the assessment programme of physical function. Of these, 41 patients had no data for HGS, QIS, or clinical outcomes, leaving 616 patients who were included in the analyses (*Figure* *S2*).

The mean age of the 616 participants was 65.4 years, 61% were men, and the median HD vintage was 2.0 years. The mean HGS was 22.8 kg, and the mean QIS was 42.5% DW. In total, 213 (34.6%) patients were classified as having either low HGS or low QIS, and 211 (34.3%) were classified as having dynapenia. Participants with dynapenia were more likely to be older, women, have lower height, DW, and BMI, higher comorbidity index score, lower levels of serum albumin, serum haemoglobin, and serum creatinine, lower number of steps, and lower HGS and QIS (*Table* 1).

**Table 1:** Patient characteristics

	Missing, *n*	Total (*N* = 616)	Robust (*N* = 192)	Either low HGS or QIS (*N* = 213)	Dynapenia (*N* = 211)
Age, years	0	65.4 (12.2)	58.8 (11.8)	66.1 (10.9)	70.7 (11.0)
Male sex, %	0	60.9%	67.7%	62.9%	52.6%
Height, cm	0	161.0 (154.0–168.0)	165.0 (157.0–171.0)	160.0 (153.5–166.5)	159.5 (150.5–165.0)
Dry weight, kg	0	55.4 (47.5–64.9)	62.0 (50.5–71.0)	54.6 (46.7–63.3)	52.7 (46.0–59.3)
Body mass index, kg/m^2^	0	22.0 (4.0)	22.8 (3.8)	21.9 (4.2)	21.5 (3.9)
Haemodialysis vintage, years	0	2.0 (0.0–8.0)	2.0 (0.0–9.5)	2.0 (0.0–8.0)	1.0 (0.0–8.0)
Primary kidney disease, %	2				
Diabetes		38.6%	29.2%	40.4%	45.5%
GN/cystic kidney disease		26.5%	34.9%	24.9%	20.4%
Hypertension		9.6%	9.9%	8.9%	10.0%
Unknown		12.8%	14.6%	13.1%	10.9%
Other		12.2%	11.5%	12.7%	12.3%
Co‐morbid conditions, %					
Atherosclerotic heart disease	2	24.3%	19.5%	23.5%	29.4%
Congestive heart failure	2	13.0%	8.9%	9.9%	19.9%
CVD/TIA	2	21.0%	12.6%	19.7%	29.9%
Diabetes	2	47.4%	36.3%	49.8%	55.0%
Co‐morbidity index, points	2	5.0 (3.0–7.0)	4.0 (2.0–6.0)	5.0 (4.0–7.0)	7.0 (4.0–9.0)
Laboratory data					
Serum albumin, g/dL	2	3.8 (0.3)	3.9 (0.3)	3.8 (0.3)	3.7 (0.3)
Serum hemoglobin, g/dL	4	10.6 (1.0)	10.9 (1.2)	10.6 (0.9)	10.5 (1.0)
Serum creatinine, mg/dL	4	10.0 (2.7)	11.4 (2.8)	9.9 (2.4)	8.9 (2.2)
C–reactive protein, mg/dL	34	0.1 (0.1–0.3)	0.1 (0.1–0.3)	0.1 (0.1–0.3)	0.1 (0.1–0.4)
Physical activity, steps	165	3717 (1902–6340)	5551 (3434–7999)	3698 (2171–5867)	2155 (986–3794)
Muscle strength					
Handgrip strength, kg	0	22.8 (8.5)	30.6 (7.6)	21.9 (5.4)	16.7 (5.8)
QIS, %dry weight	0	42.5 (14.2)	54.7 (10.4)	44.8 (10.7)	29.1 (7.3)

CVA/TIA, cerebrovascular accident/transient ischemic attack; GN, glomerulonephritis; QIS, quadriceps isometric strength.

Data are presented as mean (standard deviation) or median (interquartile range) for continuous measures, and % for categorical measures.

During follow‐up [median, 3.0 years (interquartile range: 1.0–6.0)], 163 (26.5%) patients died of the following causes: CV (*n* = 50), cancer (*n* = 14), respiratory (*n* = 21), cerebrovascular (*n* = 10), infection (*n* = 10), other (*n* = 23), and unknown (*n* = 35). A total of 288 CV hospitalizations occurred, including the following: angina (*n* = 13), acute myocardial infarction (*n* = 18), cardiac arrest (*n* = 2), congestive heart failure (*n* = 77), cardiomyopathy (*n* = 2), valvular heart disease (*n* = 15), arrhythmia (*n* = 21), pericarditis and/or tamponade (*n* = 3), thoracic aortic aneurysm and dissection (*n* = 12), coronary angioplasty (*n* = 68), coronary artery bypass graft (*n* = 13), valve repair or replacement (*n* = 7), pacemaker placement or replacement of pacemaker battery (*n* = 11), and other cardiac or CV diseases (*n* = 26).

### Association of handgrip strength and quadriceps isometric strength with outcomes

**Figure 2:**
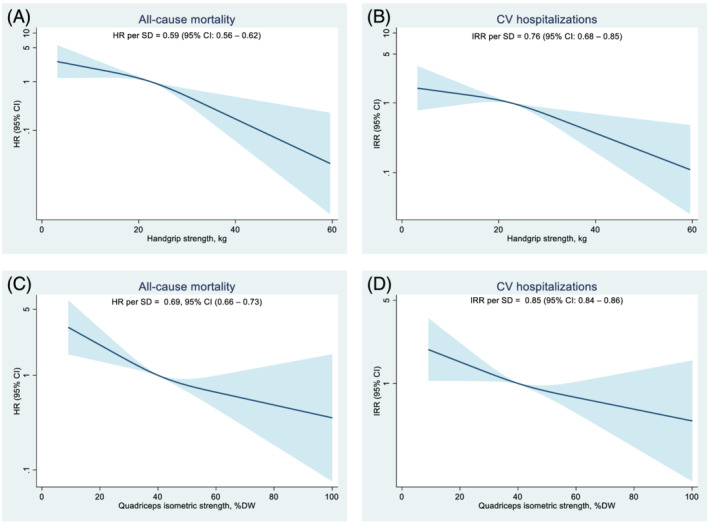
Associations of handgrip strength and quadriceps isometric strength with all‐cause mortality and cardiovascular hospitalizations. *(A)* Handgrip strength and all‐cause mortality. *(B)* Handgrip strength and cardiovascular hospitalizations. *(C)* Quadriceps isometric strength and all‐cause mortality. *(D)* Quadriceps isometric strength and cardiovascular hospitalizations. Each spline model was adjusted for age, sex, height, dry weight, and haemodialysis vintage. The reference value of handgrip strength is 22.5 kg, and quadriceps isometric strength is 40.0% DW. The *y*‐axis is a log scale with the shaded area representing 95% confidence intervals. HR (95% CI) and IRR (95% CI) were presented for the associations between per 1 SD increase of handgrip strength and quadriceps isometric strength with all‐cause mortality and CV hospitalizations. SD: 8.5 kg for handgrip strength and 14.2% DW for quadriceps isometric strength. 95% CI, 95% confidence interval; CV, cardiovascular; DW, dry weight; HR, hazard ratio; IRR, incidence rate ratio; SD, standard deviation.

When HGS and QIS were considered as the categorical models, after adjusting for potential confounders, the group with low HGS (HR: 2.11, 95% CI: 2.04–2.18, IRR: 1.63, 95% CI: 1.32–2.01) and the group with low QIS (HR: 1.66, 95% CI: 1.25–2.19, IRR: 1.57, 95% CI: 1.30–1.90) had a higher risk of both outcomes than the robust group (*Table* 2). Restricted cubic splines showed linear associations of HGS and QIS with all‐cause mortality and CV hospitalizations (*Figure* 2). When considering HGS and QIS as continuous variables, increasing HGS per 1 standard deviation (SD) (HR: 0.59, 95% CI: 0.56–0.62, IRR: 0.76, 95% CI: 0.68–0.85) and increasing QIS per 1 SD (HR: 0.69, 95% CI: 0.66–0.73, IRR: 0.85, 95% CI: 0.84–0.86) were associated with lower risks of all‐cause mortality and CV hospitalizations, respectively. Also, the continuous and categorized association of HGS and QIS with all‐cause mortality and CV hospitalizations showed similar associations in both sex‐stratified models (*Table* *S1*, *Figure* *S3*).

**Table 2:** Association of handgrip strength and quadriceps isometric strength with outcomes

	Person‐years	All‐cause mortality		CV hospitalizations	
		No. of deaths	HR [95% CI]	No. of events	IRR [95% CI]
**HGS category**					
Robust	1355	34	Reference	140	Reference
Low HGS	1522	129	2.11 [2.04–2.18]	311	1.63 [1.32–2.01]
**QIS category**					
Robust	1637	62	Reference	193	Reference
Low QIS	1240	101	1.66 [1.25–2.19]	258	1.57 [1.30–1.90]

95% CI, 95% confidence interval; CV, cardiovascular; HGS, handgrip strength; HR, hazard ratio; IRR, incidence rate ratio; QIS, quadriceps isometric strength.

Adjusted for age, sex, height, dry weight, haemodialysis vintage, comorbidity index, serum albumin, serum haemoglobin, serum creatinine, and C‐reactive protein. Low handgrip strength was defined as <28 kg for men and <18 kg for women. Low quadriceps isometric strength was defined as <40% dry weight.

### Association between dynapenia and outcomes

**Figure 3:**
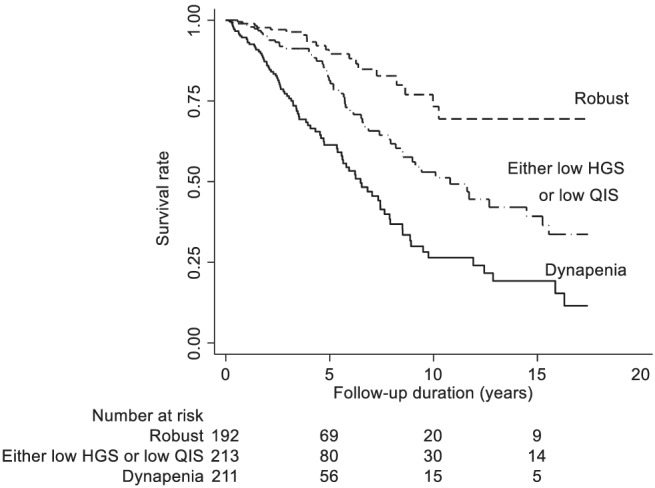
Results of Kaplan–Meier analyses for the incidence of death in 616 patients undergoing haemodialysis. Patients were divided into three groups: robust (‘high HGS and high QIS’), either low HGS or low QIS (‘low HGS and high QIS’ or ‘high HGS and low QIS’), and dynapenia (‘low HGS and low QIS’). HGS, handgrip strength; QIS, quadriceps isometric strength.

*Figure* 3 shows the Kaplan–Meier survival analysis between the robust, either low HGS or low QIS, and dynapenia groups. The log‐rank test showed significantly worse survival in the either low HGS or low QIS and dynapenia groups than in the robust group (*P* < 0.001).

*Table* 3 shows the association of dynapenia with all‐cause mortality and CV hospitalizations. After adjusting for confounders, the either low HGS or low QIS (HR: 1.75, 95% CI: 1.46–2.10, IRR: 1.41, 95% CI: 1.00–1.99) and dynapenia (HR: 2.80, 95% CI: 2.49–3.14, IRR: 2.04, 95% CI: 1.44–2.89) groups were significantly associated with higher risks of all‐cause mortality and CV hospitalizations than the robust group.

**Table 3:** Association of dynapenia with all‐cause mortality and cardiovascular hospitalizations

	Person‐years	All‐cause mortality			CV hospitalizations		
		No. of deaths	Unadjusted	Adjusted	No. of events	Unadjusted	Adjusted
			HR [95% CI]	HR [95% CI]		IRR [95% CI]	IRR [95% CI]
Robust	951	20	Reference	Reference	90	Reference	Reference
Either low HGS or QIS	1089	56	2.37 [1.85–3.04]	1.75 [1.46–2.10]	153	1.64 [1.52–1.78]	1.41 [1.00–1.99]
Dynapenia	836	87	5.17 [5.04–5.31]	2.80 [2.49–3.14]	208	2.96 [2.85–3.06]	2.04 [1.44–2.89]
*P* for trend^a^	—	—	<0.001	<0.001	—	<0.001	<0.001

95% CI, 95% confidence interval; CV, cardiovascular; HGS, handgrip strength; HR, hazard ratio; IRR, incidence rate ratio; QIS, quadriceps isometric strength.

Adjusted for age, sex, height, dry weight, haemodialysis vintage, comorbidity index, serum albumin, serum haemoglobin, serum creatinine, and C‐reactive protein.

^a^ Trend tests were performed by treating categorical variables as continuous variables.

In sensitivity analyses, the associations between dynapenia and both outcomes were martially the same when adjusting for physical activity in addition to the adjustment of variables included in *Table* 3 (*Table* *S2*). Furthermore, dynapenia was consistently associated with higher risks of all‐cause mortality and CV hospitalizations in both men and women (*Tables* *S3* and *S4*).

## Discussion

In this study, we investigated the association of dynapenia (defined as both low HGS and low QIS) with CV hospitalizations and all‐cause mortality among Japanese patients undergoing HD. We found that patients with either low HGS or low QIS had higher risks for CV hospitalizations and death, and those with low HGS and low QIS had even higher risks of the aforementioned outcomes in this population. To the best of our knowledge, this is the first study to assess muscle strength in the upper and lower extremities and examine its association with all‐cause mortality and CV hospitalizations.

The present study showed that dynapenia was associated with a higher mortality risk. Although previous studies used other definitions, our results are consistent with those of previous studies. For example, a prospective study in 187 patients on HD in France, which defined dynapenia as muscle strength below the median of QIS, showed that those with dynapenia had a higher mortality risk than those without (HR, 2.99; 95% CI, 1.18–7.61).^27^ Moreover, a meta‐analysis of six prospective studies among patients undergoing HD showed the HR of all‐cause mortality of low HGS patients was 1.88 (95% CI, 1.51–2.33) compared with high HGS.^8^ Our study expanded upon the findings of previous studies that show that a decline in either HGS or QIS indicates a poor prognosis, and combining low HGS and low QIS further worsens the prognosis. Therefore, our results suggest that measuring HGS and QIS combined is a more useful management strategy for risk stratification of patients undergoing HD.

This study also showed that dynapenia was associated with a higher risk of CV hospitalizations among patients on HD. Our findings are similar to those of several previous reports that low muscle strength is associated with higher CV event risks in patients with ESRD.^13^, ^28^ Although the underlying mechanism between muscle strength and CV events has not been explored in‐depth, several mechanisms may be involved. Low muscle strength leads to a reduction in muscle contraction‐induced factors, such as myokines. Myokines have anti‐inflammatory effects, and inflammation is associated with the development of vascular calcification and endothelial dysfunction.^29^, ^30^ Thus, the relative paucity of myokines in dynapenia may increase the risk of CV events. Another possible mechanism is that physical activity may mediate the association between dynapenia and CV hospitalizations. Low muscle strength is known to cause physical inactivity.^26^ Low levels of physical activity result in an increase in the risk of CV diseases through an increase in blood pressure, insulin resistance, and reduction in high‐density lipoprotein cholesterol.^31^, ^32^

The present study showed that dynapenia was associated with a higher prevalence of diabetes, congestive heart failure, cerebrovascular disease, and higher points of comorbidity score. These results may imply that patients on HD with dynapenia are one of the phenotypes reflecting various aetiologies such as multiple morbidities. Previous studies in populations with diabetes, congestive heart failure, and cerebrovascular disease have already revealed that these diseases cause muscle weakness.^33^, ^34^, ^35^ However, to the best of our knowledge, this is the first study to show that those who have multiple comorbidities are more likely to have dynapenia among HD populations, which is an important finding.

Muscle strength is a modifiable factor, and meta‐analysis studies have reported that exercise (e.g. aerobic and resistance exercises) significantly improves muscle strength among patients on HD.^36^, ^37^ Furthermore, a randomized control trial of 227 patients undergoing HD in Italy reported that patients who completed the home‐based exercise programme for 6 months had a lower risk of hospitalization.^38^ These findings indicate that improving muscle strength through appropriate interventions (e.g. rehabilitation) may help to reduce the risk of adverse clinical outcomes among patients on HD. Habitual screening of dynapenia in daily practice is important for intervention in this population.

Our study has several strengths. First, we used two types of muscle strength (HGS and QIS), which were objectively measured, reliable, and validated. Second, we treated CV hospitalizations as recurrent events and investigated their association with dynapenia; thus, we could more comprehensively identify CV events in the HD population.

The limitations of this study need to be acknowledged as well. First, our study was conducted only in Japanese patients undergoing HD at two dialysis facilities. Caution should be exercised when applying our results to other ethnic populations. Second, this study included only patients for whom both HGS and QIS could be evaluated, and we excluded patients who have started dialysis treatment for <3 months or those with severe limitations (i.e. unstable medical conditions). Mortality is much higher soon after initiation of HD treatment,^39^ and patients with unstable medical conditions are likely to have less muscle strength than those included in the present study. Therefore, the prevalence of dynapenia in this study may be underestimated compared with that in the whole HD population. Further studies are needed to determine whether the present results are applicable to these patients. Third, some misclassification biases of exposure cannot be ignored. In most patients, muscle strength was assessed pre‐dialysis, but some were assessed post‐dialysis or on non‐dialysis days, and the timing of evaluations was not consistent across patients. Considering that the degree of accumulation of substances (such as potassium and hydrogen ions) that may affect muscle contraction varies pre‐dialysis and post‐dialysis, we cannot rule out the possibility that the timing of the evaluation affected the muscle strength values. Moreover, we evaluated muscle strength at a single point only (baseline only). As muscle strength is a time‐dependent parameter, this may have led to misclassification bias. Nevertheless, given the present cohort design, these misclassification biases of exposure tend towards the null associations (i.e. conservative associations). Finally, we could not collect data on neurological, psychological, and dietary habits. The lack of these covariates may have led to an overestimation of the association between dynapenia and clinical outcomes in the present study.

In conclusion, dynapenia, defined by both low HGS and low QIS, was significantly associated with a higher risk of all‐cause mortality and CV hospitalizations among patients on HD. Assessing muscle strength in clinical practice and screening patients with low muscle strength are important for disease management in this population. Measuring muscle strength of the entire extremity, not only the upper or lower extremities, may be necessary for accurate prognostic stratification.

## Conflict of interest

All authors have no conflict of interest to declare.

## Supporting information




**Figure S1.** Sex‐different distributions of handgrip strength (**a**) and quadriceps isometric strength (**b**). DW, dry weight.
**Figure S2** Flow diagram of patient selection and exclusion process
**Figure S3.** Sex‐specific associations of handgrip strength and quadriceps isometric strength with all‐cause mortality and cardiovascular hospitalizations. Each spline model was adjusted for age, sex, height, dry weight, and hemodialysis vintage. The reference value of handgrip strength is 28.1 kg for men and 18.0 kg for women, and quadriceps isometric strength is 40.0% DW for men and 40.2%DW for women. The y‐axis is a log scale with the shaded area representing 95% confidence intervals. HR (95% CI) and IRR (95% CI) were presented for the associations between per 1 SD increase of handgrip strength and quadriceps isometric strength with all‐cause mortality and CV hospitalizations. SD: handgrip strength is 7.8 kg for men and 5.7 kg for women, quadriceps isometric strength is 13.8% for men and 13.4% DW for women. CV; cardiovascular; DW, dry weight; HR, hazard ratio; IRR, incidence rate ratio; SD, standard deviation; 95% CI, 95% confidence interval.
**Table S1**: Association of handgrip strength and quadriceps isometric strength with outcomes stratified according to sex
**Table S2**: Association between dynapenia and outcomes
**Table S3**: Association between dynapenia and all‐cause mortality stratified according to sex
**Table S4**: Association between dynapenia and cardiovascular hospitalizations stratified according to sex
