# Supplementary material for: Associations between dynapenia, cardiovascular hospitalizations, and all‐cause mortality among patients on haemodialysis
Source: J Cachexia Sarcopenia Muscle. 2022 Aug 2;13(5):2417–25. doi: 10.1002/jcsm.13039 (PMC9530547; doi:10.1002/jcsm.13039)
Supplement: Supplementary file 1 — Figure S1. Sex‐different distributions of handgrip strength (a) and quadriceps isometric strength (b). DW, dry weight. Figure S2 Flow diagram of patient selection and exclusion process Figure S3. Sex‐specific associations of handgrip strength and quadriceps isometric strength with all‐cause mortality and cardiovascular hospitalizations. Each spline model was adjusted for age, sex, height, dry weight, and hemodialysis vintage. The reference value of handgrip strength is 28.1 kg for men and 18.0 kg for women, and quadriceps isometric strength is 40.0% DW for men and 40.2%DW for women. The y‐axis is a log scale with the shaded area representing 95% confidence intervals. HR (95% CI) and IRR (95% CI) were presented for the associations between per 1 SD increase of handgrip strength and quadriceps isometric strength with all‐cause mortality and CV hospitalizations. SD: handgrip strength is 7.8 kg for men and 5.7 kg for women, quadriceps isometric strength is 13.8% for men and 13.4% DW for women. CV; cardiovascular; DW, dry weight; HR, hazard ratio; IRR, incidence rate ratio; SD, standard deviation; 95% CI, 95% confidence interval. Table S1: Association of handgrip strength and quadriceps isometric strength with outcomes stratified according to sex Table S2: Association between dynapenia and outcomes Table S3: Association between dynapenia and all‐cause mortality stratified according to sex Table S4: Association between dynapenia and cardiovascular hospitalizations stratified according to sex [file JCSM-13-2417-s001.docx]

Table S1: Association of handgrip strength and quadriceps isometric strength with outcomes stratified according to sex

|  | | Men | | | | | |  | Women | | | | | |
| --- | --- | --- | --- | --- | --- | --- | --- | --- | --- | --- | --- | --- | --- | --- |
|  |  | Person-years | All-cause mortality | |  | CV hospitalizations | |  | Person-years | All-cause mortality | |  | CV hospitalizations | |
|  |  |  | No. of deaths | HR [95% CI] |  | No. of events | IRR [95% CI] |  |  | No. of deaths | HR [95% CI] |  | No. of events | IRR [95% CI] |
| **HGS category** | | | | | | | | | | | | | | |
|  | Robust | 648 | 13 | Reference |  | 65 | Reference |  | 707 | 21 | Reference |  | 75 | Reference |
|  | Low HGS | 843 | 77 | 3.02 [2.03 – 4.48] |  | 184 | 1.83 [1.34 – 2.52] |  | 678 | 52 | 1.63 [1.56 – 1.71] |  | 127 | 1.46 [1.39 – 1.55] |
| **QIS category** | | | | | | | | | | | | | | |
|  | Robust | 1040 | 49 | Reference |  | 140 | Reference |  | 597 | 13 | Reference |  | 53 | Reference |
|  | Low QIS | 451 | 41 | 1.45 [1.15 – 1.83] |  | 109 | 1.51 [1.14 – 2.00] |  | 789 | 60 | 2.24 [1.93 – 2.60] |  | 149 | 1.68 [1.60 – 1.76] |
| CV, cardiovascular; HGS, handgrip strength; HR, hazard ratio; IRR, incidence rate ratio; QIS, quadriceps isometric strength; 95% CI, 95% confidence interval. Adjusted for age, height, dry weight, hemodialysis vintage, comorbidity index, serum albumin, serum hemoglobin, serum creatinine, and C-reactive protein. Low handgrip strength was defined as <28 kg for men and <18 kg for women. Low quadriceps isometric strength was defined as <40% dry weight. | | | | | | | | | | | | | | |

Table S2: Association between dynapenia and outcomes

|  | Person-years | All-cause mortality | |  | CV hospitalizations | |
| --- | --- | --- | --- | --- | --- | --- |
|  |  | No. of deaths | HR [95% CI] |  | No. of events | IRR [95% CI] |
| Robust | 951 | 20 | Reference |  | 90 | Reference |
| Either low HGS or low QIS | 1089 | 56 | 1.68 [1.37 – 2.07] |  | 153 | 1.36 [0.99 – 1.88] |
| Dynapenia | 836 | 87 | 2.55 [2.27 – 2.88] |  | 208 | 1.87 [1.38 – 2.53] |
| *P* for trend* | – | – | <0.001 |  | – | <0.001 |
| CV, cardiovascular; HGS, handgrip strength; HR, hazard ratio; IRR, incidence rate ratio; QIS, quadriceps isometric strength; 95% CI, 95% confidence interval. Adjusted for age, sex, height, dry weight, hemodialysis vintage, comorbidity index, serum albumin, serum creatinine, serum hemoglobin, C-reactive protein, and physical activity. *Trend tests were performed by treating categorical variables as continuous variables. | | | | | | |

Table S3: Association between dynapenia and all-cause mortality stratified according to sex

| All-cause mortality | Men (N=375) | | | |  | | Women (N=241) | | | | |  |
| --- | --- | --- | --- | --- | --- | --- | --- | --- | --- | --- | --- | --- |
|  | N | Person-years | No. of deaths | HR [95% CI] | |  | | N | Person-years | No. of deaths | HR [95% CI] | |
| Robust | 130 | 574 | 12 | Reference | |  | | 62 | 378 | 8 | Reference | |
| Either low HGS or low QIS | 134 | 540 | 38 | 2.41 [1.37 – 4.23] | |  | | 79 | 549 | 18 | 1.15 [1.01 – 1.30] | |
| Dynapenia | 111 | 378 | 40 | 3.11 [2.52 – 3.84] | |  | | 100 | 459 | 47 | 2.44 [1.96 – 3.03] | |
| *P* for trend* | – | – | – | <0.001 | |  | | – | – | – | <0.001 | |
| HGS, handgrip strength; HR, hazard ratio; QIS, quadriceps isometric strength; 95% CI, 95% confidence interval. Adjusted for age, height, dry weight, hemodialysis vintage, comorbidity index, serum albumin, serum creatinine, serum hemoglobin, and C-reactive protein. *Trend tests were performed by treating categorical variables as continuous variables. | | | | | | | | | | | |  |

Table S4: Association between dynapenia and cardiovascular hospitalizations stratified according to sex

| CV hospitalizations | Men (N=375) | | | |  | Women (N=241) | | | |
| --- | --- | --- | --- | --- | --- | --- | --- | --- | --- |
|  | N | Person-years | No. of events | IRR [95% CI] |  | N | Person-years | No. of events | IRR [95% CI] |
| Robust | 130 | 574 | 56 | Reference |  | 62 | 378 | 34 | Reference |
| Either low HGS or low QIS | 134 | 540 | 93 | 1.54 [1.15 – 2.06] |  | 79 | 549 | 60 | 1.24 [0.87 – 1.76] |
| Dynapenia | 111 | 378 | 100 | 2.10 [1.33 – 3.31] |  | 100 | 459 | 108 | 1.94 [1.64 – 2.30] |
| *P* for trend* | – | – | – | 0.001 |  | – | – | – | <0.001 |
| CV, cardiovascular; HGS, handgrip strength; IRR, incidence rate ratio; QIS, quadriceps isometric strength; 95% CI, 95% confidence interval. Adjusted for age, height, dry weight, hemodialysis vintage, comorbidity index, serum albumin, serum creatinine, serum hemoglobin, and C-reactive protein. *Trend tests were performed by treating categorical variables as continuous variables. | | | | | | | | | |


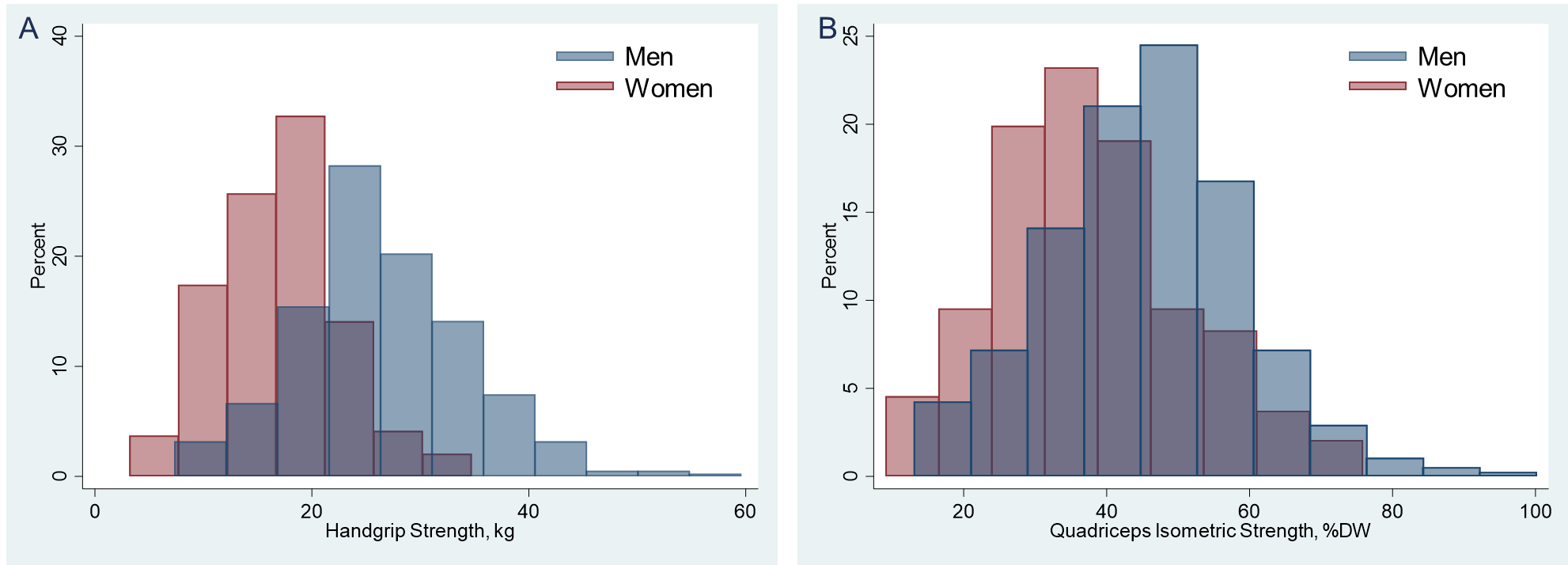


**Fig. S1.** Sex-different distributions of handgrip strength (A) and quadriceps isometric strength (B).

DW, dry weight.

**
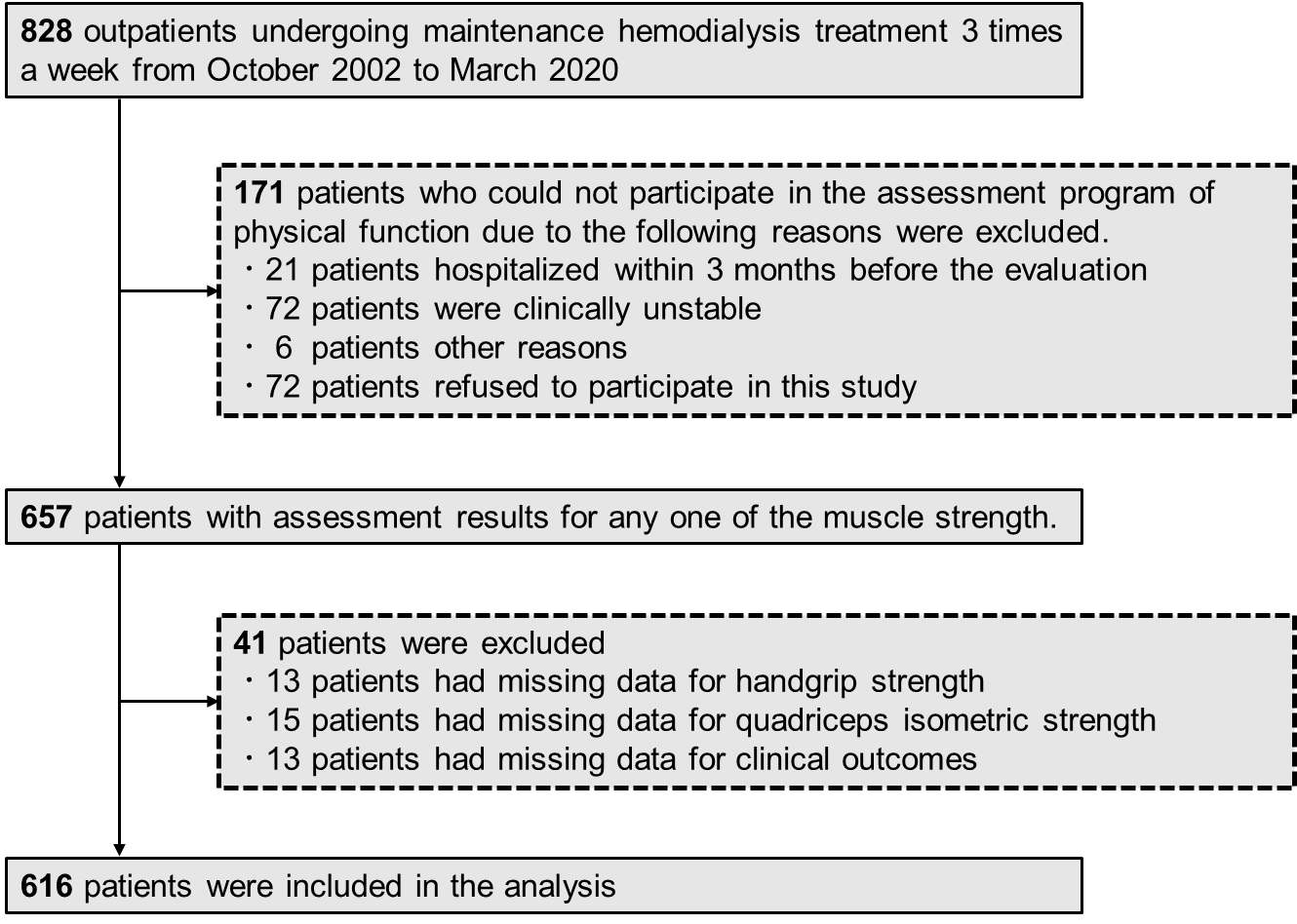
**

**Fig. S2.** Flow diagram of patient selection and exclusion process


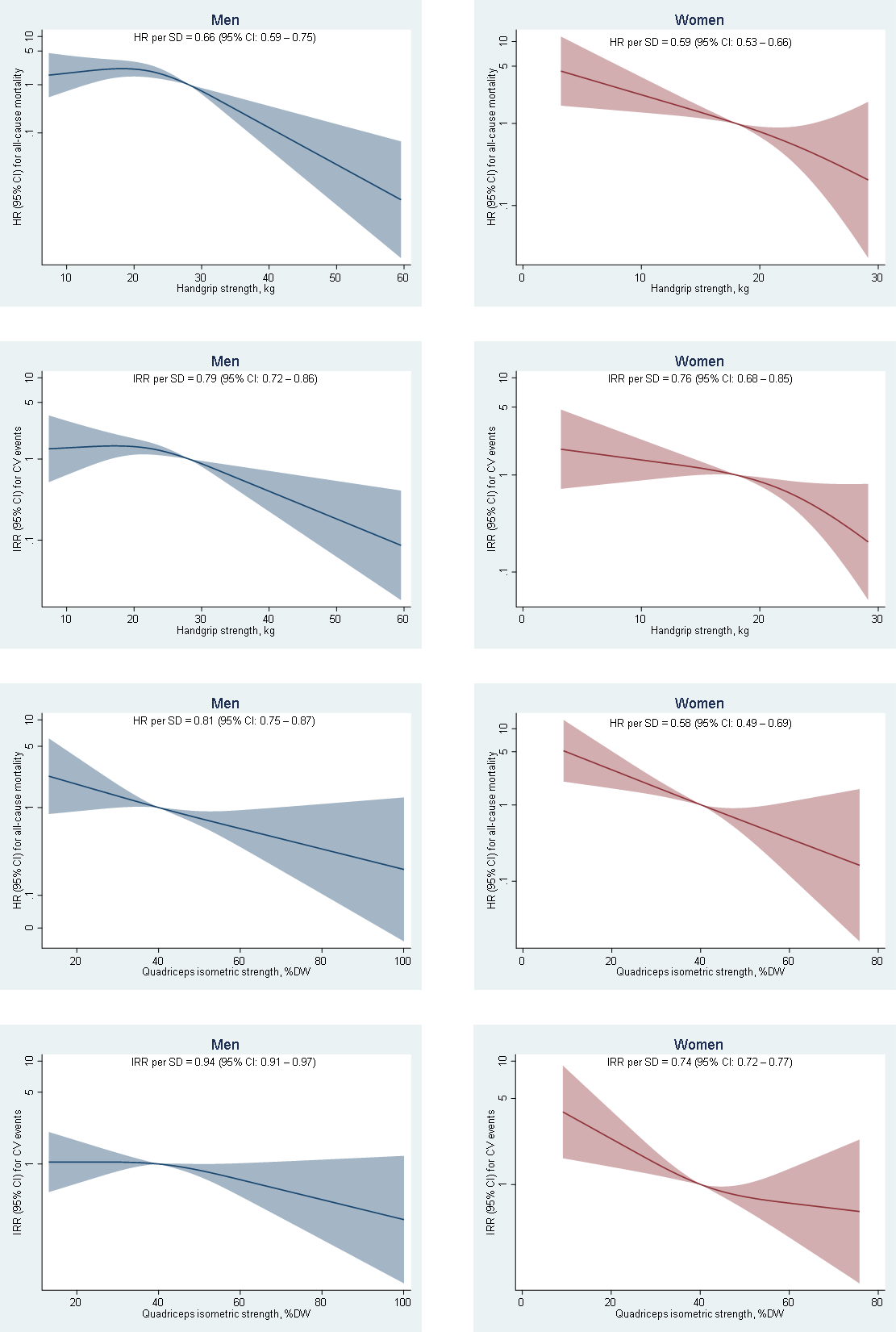


**Fig. S3.** Sex-specific associations of handgrip strength and quadriceps isometric strength with all-cause mortality and cardiovascular hospitalizations.

Each spline model was adjusted for age, sex, height, dry weight, and hemodialysis vintage. The reference value of handgrip strength is 28.1 kg for men and 18.0 kg for women, and that of quadriceps isometric strength is 40.0% DW for men and 40.2% DW for women. The y-axis is a log scale with the shaded area representing 95% confidence intervals. HR (95% CI) and IRR (95% CI) were presented for the associations between per 1 SD increase of handgrip strength and quadriceps isometric strength with all-cause mortality and CV hospitalizations. SD: handgrip strength is 7.8 kg for men and 5.7 kg for women, quadriceps isometric strength is 13.8% DW for men and 13.4% DW for women. CV; cardiovascular; DW, dry weight; HR, hazard ratio; IRR, incidence rate ratio; SD, standard deviation; 95% CI, 95% confidence interval.
